# Supplementary material for: The interactome of intact mitochondria by cross-linking mass spectrometry provides evidence for coexisting respiratory supercomplexes
Source: Mol Cell Proteomics. 2017 Dec 8;17(2):216–32. doi: 10.1074/mcp.RA117.000470 (PMC5795388; doi:10.1074/mcp.RA117.000470)
Supplement: Supplemental Data [file supp_RA117.000470_133922_0_supp_23978_fzffwf.zip › spectra_annotation/mito_DR_spectra_annotation/98-1-1-1-10-1.pdf]

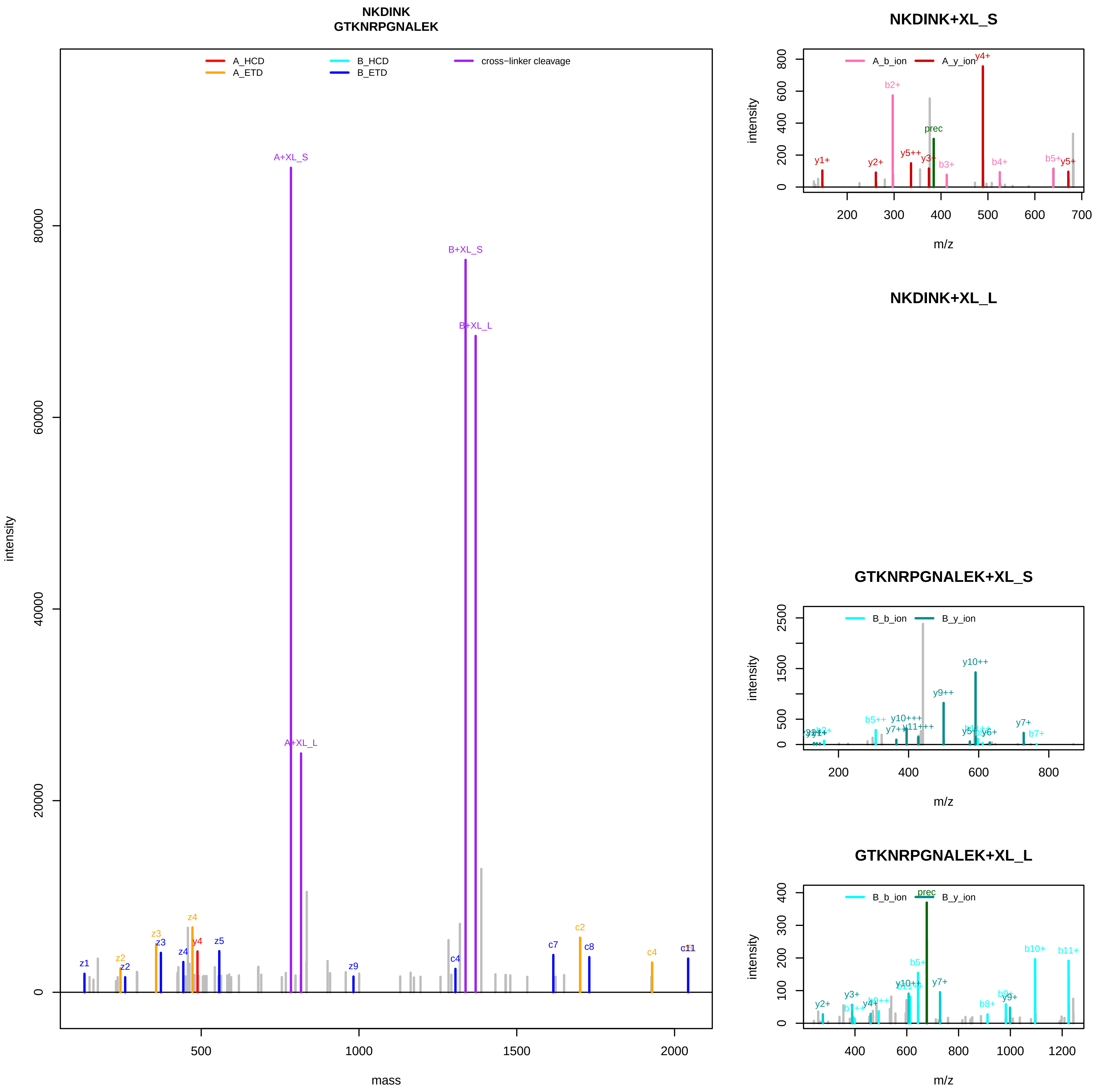
NKDINK
GTKNRPGNALEK
A_HCD
A_ETD
B_HCD
B_ETD
cross−linker cleavage
A+XL_S
B+XL_S
B+XL_L
A+XL_L
z1
z2
z3
z4
y4
z5
z9
c4
c7
c2
c8
c11
intensity
mass
0
20000
40000
60000
80000
500
1000
1500
2000
NKDINK+XL_S
A_b_ion
A_y_ion
y4+
b2+
prec
y1+
y2+
y5++
y3+
b3+
b4+
b5+
y5+
m/z
NKDINK+XL_L
GTKNRPGNALEK+XL_S
B_b_ion
B_y_ion
y10++
y9++
b5++
y10+++
y7++
y11+++
y7+
y6+
b7+
GTKNRPGNALEK+XL_L
prec
b10+
b11+
b5+
y10++
y7+
y3+
y2+
b8+
b9+
y9+
